# Evaluating the link between the dietary intake of vitamin B and constipation: a population-based study

**DOI:** 10.3389/fnut.2025.1594644

**Published:** 2025-05-29

**Authors:** Wenyuan Yang, Chenyu Jiang, Yaojian Shao, Shicheng Gu, Mingqi Hong

**Affiliations:** ^1^Department of Gastroenterology, Taizhou Central Hospital (Taizhou University Hospital), Taizhou, Zhejiang, China; ^2^Department of Geriatric, Taizhou Central Hospital (Taizhou University Hospital), Taizhou, Zhejiang, China

**Keywords:** constipation, vitamin B, dietary intake, NHANES, population-based study

## Abstract

**Background:**

Prior research has established a correlation between dietary micronutrient intake and the risk of developing constipation. However, the potential link between vitamin B intake and constipation has yet to be fully explored. This study sought to assess the association between chronic constipation and dietary vitamin B intake based on National Health and Nutrition Examination Survey (NHANES) data.

**Methods:**

This study employed NHANES data collected between 2005 and 2010, including a total of 13,885 participants 20 + years of age. Dietary intake of vitamins B1, B2, niacin, B6, folic acid, choline, and B12 was assessed using the first 24-h dietary recall interview. Constipation was defined based on the Bristol Stool Form Scale (BSFS). A weighted logistic regression model and restricted cubic spline (RCS) analysis were utilized to adjust for demographic and lifestyle-related variables and probe the relationship between B vitamin intake and constipation. Statistical significance was set at a two-tailed *p*-value < 0.05. Confidence intervals (95% CIs) were calculated using weighted logistic regression.

**Results:**

The analysis revealed a nonlinear inverse correlation between dietary intake of all examined B vitamins and constipation risk. In particular, a significant reduction in constipation odds was observed in the highest intake quartiles of niacin (OR = 0.76, 95% CI: 0.59–0.99, p for trend = 0.003), folate (OR = 0.61, 95% CI: 0.48–0.79, p for trend < 0.001), and choline (OR = 0.77, 95% CI: 0.60–1.00, p for trend = 0.05) when using a model that was fully adjusted. Subgroup analyses further indicated that alcohol consumption significantly modified the relationship between folate (p for interaction = 0.003), vitamin B1 (p for interaction = 0.004), niacin (p for interaction = 0.04), choline (p for interaction = 0.02), and constipation.

**Conclusion:**

Increased dietary intake of B vitamins may contribute to a reduced risk of constipation, particularly among specific population subgroups. These results offer additional support for the potential role of dietary modifications in promoting gut health.

## 1 Introduction

Constipation is a widespread gastrointestinal disorder with significant effects on quality of life that imposes a considerable healthcare burden (1). Infrequent bowel movements, difficulty passing stools, and a feeling of incomplete evacuation are all hallmark features of constipation (2). Epidemiological studies estimate that among adults, constipation has a prevalence of approximately 10.1%, with higher rates observed among older individuals and women (3). Clinically, constipation is generally separated into three subtypes: slow-transit constipation, normal-transit constipation, and pelvic floor dysfunction (4). Several primary risk factors have been identified, including dietary habits, socioeconomic status, lifestyle choices, levels of physical activity, medication usage, and mental health conditions such as depression, all of which have been closely linked to constipation (5). Among these, diet represents a modifiable factor, making it a key area of interest for both constipation prevention and management. Previous studies have suggested that inadequate fluid intake and excessive consumption of dietary saturated fat and energy may increase constipation risk, whereas a high intake of soluble fiber, selenium, and an overall high-quality diet may reduce it (6–9). These findings emphasize the utility of dietary regulation as an effective means of managing constipation.

B vitamins are water-soluble vitamins commonly found in various foods. Key forms include vitamin B1 (thiamine), B2 (riboflavin), B3 (niacin), B6 (pyridoxine), B9 (folate), choline, and B12 (cobalamin). These vitamins play essential roles in human growth and metabolism. Since the human body cannot synthesize B vitamins autonomously, they must be obtained through dietary intake, with a minor contribution from gut microbiota synthesis. Functionally, B vitamins serve as cofactors and coenzymes in multiple metabolic pathways, playing crucial roles in cellular energy metabolism, signal transduction, biosynthesis of bioactive compounds, and immune system regulation. Additionally, they are vital for neurological function and digestive system health (10–13). Theoretically, B vitamins may be able to influence intestinal motility by modulating neural control of gut muscles, regulating immune metabolism, and mitigating intestinal inflammation. However, the specific link between B vitamin consumption and constipation remains largely unexplored.

Most existing studies probing the interplay between B vitamins and constipation have been limited to small-scale clinical trials (14, 15), which often suffer from small sample sizes and insufficient data analysis methodologies. Large-scale clinical databases offer an opportunity to obtain more robust and generalizable findings. This study leveraged NHANES data to retrospectively analyze the link between dietary intake of various B vitamins and constipation among adults, with the *a priori* hypothesis that increased B-vitamin consumption would demonstrate an inverse relationship with constipation occurrence. The aim of the results is to provide precise nutritional guidance for the prevention and management of constipation, essentially on dietary interventions, and precisely using B vitamin intake.

## 2 Methods

### 2.1 Data source

The NHANES study, conducted by the National Center for Health Statistics (NCHS), is a nationally representative program designed to assess nutritional status and health among members of the population of the USA. Data collection follows a stratified, multistage sampling methodology, and the study protocol has received approval from the NCHS Ethics Review Board, with written informed consent from all enrolled participants. For the present study, NHANES data from 2005 to 2010 were analyzed, as these three 2-year cycles included relevant questionnaire data on bowel health. Eligible participants were at least 20 years old, had completed the Bowel Health Questionnaire (BHQ), and had a valid first 24-h dietary recall. A total of 14,374 participants met these criteria. However, individuals who were pregnant (*n* = 407) or reported using laxatives (*n* = 82) were excluded from the final analyses. This led to a study population of 13,885 individuals.

### 2.2 Evaluation of dietary B vitamin intake

NHANES employs two 24-h dietary recall interviews to assess participants’ dietary intake. Initial analyses are performed in person at a Mobile Examination Center (MEC), while the second is performed via telephone 3–10 days later. Due to missing data in the second dietary recall, the present study relied on the reported dietary intake from the first 24-h recall interview. The intake of various B vitamins, including vitamins B1, B2, niacin, B6, folate, choline, and B12, was assessed based on these reports.

### 2.3 Definition of constipation

Previous research has established that constipation can be evaluated with the Bristol Stool Form Scale (BSFS), which is included in the BHQ. The BSFS provides detailed descriptions and corresponding color-coded cards representing seven distinct stool types. Individuals were instructed to examine the card and select the stool type that best represented their usual or most common bowel movements. Consistent with prior NHANES studies, stool types 1 (separate hard lumps, resembling nuts) and 2 (sausage-shaped, lumpy) were classified as indicative of constipation, whereas types 3–7 were considered representative of normal bowel habits (9).

### 2.4 Study covariates

Demographic and lifestyle data for this study were obtained through standardized questionnaires administered during initial interviews. The demographic variables included age (years), gender (male, female), ethnicity (Non-Hispanic Black, Non-Hispanic White, Mexican American, Other Hispanic, and Other), family poverty-to-income ratio (PIR), educational attainment (< high school, high school, > high school), and body mass index (BMI, kg/m^2^). Lifestyle variables encompassed recreational activity (or muscle-strengthening exercise), smoking status, alcohol consumption, and overall dietary quality. Recreational activity was categorized into two groups: individuals who engaged in no recreational activity or muscle-strengthening exercises and those who participated in such activities, regardless of intensity or duration. Smoking status was classified as non-smoking (including former and never smokers) and current smoking. Alcohol consumption was categorized as non-drinking (including former drinkers and those who never consumed alcohol) and current drinking. Overall dietary quality was assessed using the Healthy Eating Index (HEI)-2015, derived from the first 24-h dietary recall. The HEI-2015 has a maximum score of 100, with higher scores reflecting superior dietary quality. Diabetes mellitus was defined based on self-reported diagnoses, current use of insulin or other antidiabetic medications, fasting blood glucose levels ≥ 126 mg/dL, HbA1c ≥ 6.5%, or a 2-h post-load serum glucose level ≥ 200 mg/dL following a 75 g oral glucose tolerance test. Hypertension was identified based on an SBP ≥ 140 mmHg, DBP ≥ 90 mmHg, self-reported diagnosis, or the use of antihypertensive medications. Depression was assessed using the 9-item Patient Health Questionnaire (PHQ-9), a validated and publicly available screening tool, with scores ≥ 10 indicating the presence of depression.

### 2.5 Statistical analyses

To accommodate the complex, multi-stage sampling design of NHANES, ‘wtdr1d’ sample weights were utilized. Categorical variables were expressed as frequencies (weighted percentages) and analyzed using chi-square tests, whereas continuous variables were presented as means ± standard error (SE) and compared with Student’s t-tests. The intake levels of vitamin B1, B2, niacin, B6, folate, choline, and B12 were stratified into quartiles (Q1, Q2, Q3, and Q4). Three weighted logistic regression models were developed to calculate the odds ratios (ORs) and 95% confidence intervals (CIs) for the relationships between B vitamin intake and constipation risk. Model 1 did not receive adjustments, while Model 2 controlled for age, sex, and ethnicity. Model 3 incorporated further adjustments for educational attainment, BMI, HEI-2015 total score, recreational activity, smoking status, alcohol consumption, diabetes mellitus, hypertension, and depression. Restricted cubic spline (RCS) analyses were performed to examine nonlinear associations between B vitamin intake and constipation. Furthermore, stratified analyses were conducted to assess the impact of various factors, including sex, ethnicity, educational level, recreational activity, smoking, alcohol intake, diabetes mellitus, hypertension, and depression. Data processing and statistical analyses were performed in R (v 4.2.2) with the NHANESR, survey, and rms packages. A two-tailed *p*-value < 0.05 was deemed significant.

## 3 Results

### 3.1 Participant characteristics

A total of 13,885 participants (weighted *n* = 194,346,342) from the NHANES 2005–2010 cycles were analyzed. Table 1 shows baseline characteristics for these participants based on constipation status. Among them, 1,043 individuals were classified as having constipation. Significant differences were detected between the constipation and non-constipation groups with respect to sex, ethnicity, educational attainment, PIR, BMI, depression, recreational activity, and alcohol consumption (*p* < 0.05). Although aging is generally considered a potential risk factor for constipation, no differences in age distribution were observed between the groups (*p* = 0.2). Regarding dietary intake, individuals with constipation reported lower consumption levels of vitamin B1, B2, niacin, B6, folate, choline, and B12, along with a tendency toward poorer dietary quality, as indicated by lower HEI-2015 scores.

**Table 1:** Participant characteristics.

	Non-constipation	Constipation	*p* value
	*N* = 12,842	*N* = 1,043	
Vitamin B1 (mg)	1.68 (0.02)	1.44 (0.04)	< 0.0001
Vitamin B2 (mg)	2.26 (0.02)	1.97 (0.05)	< 0.0001
Niacin (mg)	26.02 (0.24)	21.90 (0.44)	< 0.0001
Vitamin B6 (mg)	2.07 (0.02)	1.75 (0.04)	< 0.0001
Folate (mcg)	415.07 (4.41)	347.49 (8.30)	< 0.0001
Choline (mg)	339.47 (2.72)	290.64 (8.19)	< 0.0001
Vitamin B12 (mcg)	6.55 (0.09)	5.47 (0.23)	< 0.0001
HEI 2015 score	50.78 (0.30)	49.25 (0.66)	0.02
Age (years)	47.12 (0.36)	46.26 (0.62)	0.2
PIR	3.11 (0.04)	2.67 (0.08)	< 0.0001
Body mass index (BMI, kg/m2)	28.77 (0.12)	27.64 (0.28)	< 0.001
Sex (%)			< 0.0001
Female	6,160 (49.46)	700 (71.10)	
Male	6,682 (50.54)	343 (28.90)	
Race (%)			0.003
Mexican American	2,299 (7.76)	188 (9.61)	
Non-Hispanic Black	2,525 (10.63)	245 (15.45)	
Non-Hispanic White	6,443 (72.41)	454 (64.24)	
Other Hispanic	1,063 (4.12)	117 (5.20)	
Other Race	512 (5.09)	39 (5.51)	
Educational status (%)			< 0.0001
Less than high school	1,488 (5.54)	155 (8.57)	
High school	5,097 (36.10)	478 (44.57)	
More than high school	6,248 (58.36)	407 (46.86)	
Diabetes mellitus (%)			0.59
No	10,537 (87.38)	871 (88.05)	
Yes	2,305 (12.62)	172 (11.95)	
Hypertension (%)			0.24
No	7,388 (62.71)	642 (65.04)	
Yes	5,452 (37.29)	401 (34.96)	
Depression (%)			< 0.0001
No	11,769 (92.79)	902 (86.76)	
Yes	1,059 (7.21)	138 (13.24)	
Recreational activity(%)			< 0.0001
No	7,581 (53.39)	681 (61.12)	
Yes	5,261 (46.61)	362 (38.88)	
Smoking status (%)			0.7
No	9,947 (76.92)	835 (77.60)	
Yes	2,892 (23.08)	208 (22.40)	
Drinking status (%)			< 0.001
No	4,151 (26.42)	426 (34.66)	
Yes	8,674 (73.58)	616 (65.34)	

PIR, Family income to poverty ratio.

### 3.2 The link between vitamin B intake and constipation

**Figure 1:**
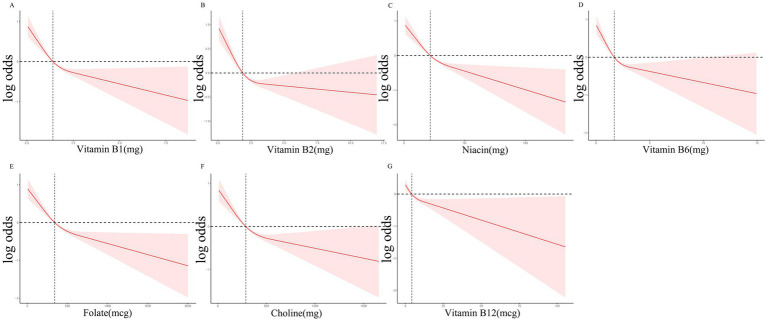
RCS curves for the associations between the B Vitamins intake and the occurrence of constipation.

Table 2 illustrates the relationship between B vitamin intake quartiles and constipation. Weighted logistic regression analysis indicated that participants in the Q3 and Q4 quartiles for all B vitamins exhibited a negative association with constipation compared to those in Q1 in Model 1 (*p* < 0.05). Similar trends were observed in Model 2 following adjustment for age, sex, and ethnicity, with significant associations for vitamin B1 (p for trend = 0.01), B2 (p for trend = 0.02), niacin (p for trend < 0.0001), B6 (p for trend = 0.002), folate (p for trend < 0.0001), and choline (p for trend = 0.002). However, no significant relationship was detected between vitamin B12 intake and constipation in Model 2 (p for trend = 0.26). Following further adjustment for educational attainment, BMI, HEI-2015 score, recreational activity, smoking status, alcohol consumption, diabetes mellitus, hypertension, and depression in Model 3, a significant reduction in constipation odds was observed in the highest intake quartiles of niacin (OR = 0.76, 95% CI: 0.59–0.99, p for trend = 0.003), folate (OR = 0.61, 95% CI: 0.48–0.79, p for trend < 0.001), and choline (OR = 0.77, 95% CI: 0.60–1.00, p for trend = 0.05). RCS analyses demonstrated a nonlinear, L-shaped relationship between vitamin B1, B2, niacin, B6, folate, choline, and B12 intake and constipation (Figure 1).

**Table 2:** Logistic regression analyses of the relationship between quartiles of B vitamins intake and constipation.

	Model 1		Model 2		Model 3	
	OR (95% CI)	*p* value	OR (95% CI)	*p* value	OR (95% CI)	*p* value
Vitamin B1						
Quartile 1	ref		ref		ref	
Quartile 2	0.79 (0.64, 0.98)	0.03	0.87 (0.70, 1.08)	0.20	0.94 (0.75, 1.18)	0.61
Quartile 3	0.51 (0.37, 0.70)	<0.0001	0.61 (0.44, 0.84)	0.004	0.67 (0.48, 0.93)	0.02
Quartile 4	0.49 (0.37, 0.67)	<0.0001	0.70 (0.52, 0.96)	0.03	0.76 (0.55, 1.05)	0.09
p for tend		<0.0001		0.01		0.03
Vitamin B2						
Quartile 1	ref		ref		ref	
Quartile 2	0.74 (0.58, 0.94)	0.01	0.83 (0.66, 1.06)	0.14	0.90 (0.71, 1.15)	0.40
Quartile 3	0.58 (0.44, 0.75)	<0.001	0.72 (0.55, 0.94)	0.02	0.80 (0.62, 1.04)	0.09
Quartile 4	0.52 (0.40, 0.67)	<0.0001	0.75 (0.58, 0.97)	0.03	0.83 (0.65, 1.07)	0.14
p for tend		<0.0001		0.02		0.11
Niacin						
Quartile 1	ref		ref		ref	
Quartile 2	0.88 (0.68, 1.12)	0.29	0.95 (0.75, 1.22)	0.7	1.02 (0.79, 1.33)	0.86
Quartile 3	0.51 (0.40, 0.64)	<0.0001	0.61 (0.48, 0.79)	<0.001	0.69 (0.53, 0.90)	0.01
Quartile 4	0.46 (0.36, 0.59)	<0.0001	0.67 (0.53, 0.84)	<0.001	0.76 (0.59, 0.99)	0.04
p for tend		<0.0001		<0.0001		0.003
Vitamin B6						
Quartile 1	ref		ref		ref	
Quartile 2	0.58 (0.48, 0.71)	<0.0001	0.65 (0.53, 0.79)	<0.001	0.71 (0.57, 0.89)	0.004
Quartile 3	0.65 (0.50, 0.86)	0.003	0.80 (0.60, 1.05)	0.11	0.90 (0.67, 1.21)	0.47
Quartile 4	0.43 (0.34, 0.53)	<0.0001	0.60 (0.47, 0.76)	<0.0001	0.70 (0.53, 0.92)	0.01
p for tend		<0.0001		0.002		0.07
Folate						
Quartile 1	ref		ref		ref	
Quartile 2	0.62 (0.47, 0.83)	0.001	0.68 (0.52, 0.90)	0.01	0.73 (0.56, 0.96)	0.03
Quartile 3	0.52 (0.40, 0.69)	<0.0001	0.63 (0.47, 0.82)	0.001	0.70 (0.52, 0.93)	0.02
Quartile 4	0.41 (0.33, 0.52)	<0.0001	0.55 (0.44, 0.69)	<0.0001	0.61 (0.48, 0.79)	<0.001
p for tend		<0.0001		<0.0001		<0.001
Choline						
Quartile 1	ref		ref		ref	
Quartile 2	0.72 (0.57, 0.90)	0.01	0.79 (0.63, 1.00)	0.05	0.87 (0.68, 1.10)	0.22
Quartile 3	0.59 (0.46, 0.76)	<0.0001	0.73 (0.58, 0.93)	0.01	0.82 (0.64, 1.04)	0.10
Quartile 4	0.46 (0.36, 0.58)	<0.0001	0.67 (0.53, 0.85)	0.001	0.77 (0.60, 1.00)	0.05
p for tend		<0.0001		0.002		0.05
Vitamin B12						
Quartile 1	ref		ref		ref	
Quartile 2	0.90 (0.71, 1.14)	0.37	1.00 (0.79, 1.28)	0.97	1.06 (0.83, 1.36)	0.62
Quartile 3	0.71 (0.55, 0.91)	0.01	0.86 (0.66, 1.12)	0.25	0.92 (0.71, 1.20)	0.53
Quartile 4	0.68 (0.52, 0.88)	0.004	0.89 (0.68, 1.17)	0.39	0.96 (0.73, 1.27)	0.79
p for tend		0.002		0.26		0.59

Model 1 had no covariate-adjusted; Model 2 adjusted age, sex, and ethnicity; Model 3 adjusted age, sex, ethnicity, educational attainment, BMI, HEI-2015 total score, recreational activity, smoking status, alcohol consumption, diabetes mellitus, hypertension, and depression.

### 3.3 Subgroup analyses

Subgroup analyses were employed to explore potential interaction effects on the association between B vitamin intake and constipation. As shown in Table 3, the inverse relationship linking folate intake and constipation remained significant across all subgroups. Moreover, alcohol consumption significantly influenced the relationship between folate intake and constipation (p for interaction = 0.003). Notably, drinking status also moderated the associations between constipation and vitamin B1 (p for interaction = 0.004, Supplementary Table S1), niacin (p for interaction = 0.04, Supplementary Table S2), and choline (p for interaction = 0.02, Supplementary Table S3).

**Table 3:** Subgroup analyses focused on the relationship between B Vitamins intake and the incidence of constipation.

Folate	Quartile 1	Quartile 2	Quartile 3	Quartile 4	p for trend	p for interaction
Sex						0.06
Female	ref	0.66 (0.49, 0.90)	0.70 (0.51, 0.96)	0.64 (0.49, 0.85)	0.01	
Male	ref	0.62 (0.36, 1.09)	0.40 (0.23, 0.69)	0.34 (0.23, 0.51)	<0.0001	
Race						0.19
Mexican American	ref	0.60 (0.33, 1.09)	0.48 (0.27, 0.87)	0.50 (0.28, 0.88)	0.02	
Non-Hispanic Black	ref	0.91 (0.58, 1.43)	0.61 (0.41, 0.91)	0.59 (0.32, 1.09)	0.02	
Non-Hispanic White	ref	0.62 (0.42, 0.92)	0.57 (0.40, 0.81)	0.40 (0.29, 0.56)	<0.0001	
Other Hispanic	ref	0.77 (0.39, 1.54)	0.77 (0.41, 1.42)	0.49 (0.26, 0.90)	0.03	
Other Race	ref	0.27 (0.09, 0.81)	0.11 (0.02, 0.48)	0.29 (0.11, 0.78)	0.05	
Educational status						0.76
Less than high school	ref	0.61 (0.29, 1.24)	0.41 (0.20, 0.86)	0.35 (0.19, 0.66)	0.004	
High school	ref	0.55 (0.36, 0.83)	0.53 (0.36, 0.78)	0.45 (0.28, 0.72)	0.002	
More than high school	ref	0.80 (0.49, 1.30)	0.63 (0.41, 0.95)	0.48 (0.32, 0.72)	<0.0001	
Diabetes mellitus						0.44
No	ref	0.65 (0.48, 0.88)	0.53 (0.39, 0.71)	0.40 (0.32, 0.51)	<0.0001	
Yes	ref	0.46 (0.26, 0.80)	0.48 (0.28, 0.82)	0.50 (0.30, 0.84)	0.02	
Hypertension						0.91
No	ref	0.65 (0.47, 0.91)	0.54 (0.39, 0.75)	0.41 (0.30, 0.56)	<0.0001	
Yes	ref	0.57 (0.39, 0.84)	0.48 (0.32, 0.73)	0.41 (0.28, 0.60)	<0.0001	
Depression						0.23
No	ref	0.63 (0.46, 0.85)	0.57 (0.42, 0.76)	0.45 (0.35, 0.58)	<0.0001	
Yes	ref	0.81 (0.50, 1.30)	0.39 (0.19, 0.81)	0.31 (0.15, 0.63)	<0.001	
Recreational activity						0.76
No	ref	0.69 (0.49, 0.96)	0.54 (0.37, 0.78)	0.45 (0.33, 0.62)	<0.0001	
Yes	ref	0.53 (0.30, 0.92)	0.52 (0.36, 0.77)	0.40 (0.27, 0.58)	<0.0001	
Smoking status						0.97
No	ref	0.62 (0.46, 0.83)	0.53 (0.40, 0.70)	0.41 (0.32, 0.52)	<0.0001	
Yes	ref	0.63 (0.39, 1.01)	0.48 (0.27, 0.84)	0.41 (0.24, 0.69)	0.001	
Drinking status						0.003
No	ref	0.84 (0.59, 1.18)	0.70 (0.47, 1.04)	0.79 (0.57, 1.09)	0.08	
Yes	ref	0.54 (0.38, 0.76)	0.46 (0.33, 0.64)	0.31 (0.22, 0.43)	<0.0001	

## 4 Discussion

This large-scale observational study, utilizing data from the NHANES database, identified a significant inverse relationship between vitamin B intake and constipation among American adults, which corroborates our priori hypothesis. The link between vitamin B intake and constipation followed an L-shaped curve for all vitamin B subclasses. Even after adjusting for multiple confounding variables, the negative correlation remained statistically significant for folate, niacin, and choline intake.

Previous clinical trials have suggested that dietary supplements containing B vitamins can alleviate abdominal symptoms and enhance bowel movements in individuals with irritable bowel syndrome (16–18). A meta-analysis conducted by Schoot et al. encompassing 16 studies concluded that high dietary fiber intake improves stool consistency and increases bowel movement frequency (19). Similarly, Panarese et al. demonstrated in a prospective study that individuals with intestinal motility disorders exhibited lower 25-hydroxyvitamin D levels compared to controls (20). Furthermore, an analysis of NHANES data by Cai et al. identified an independent negative correlation between vitamin E intake and constipation incidence among American adults (21). These results underscore the importance of considering multiple dietary components, including B vitamins, when evaluating chronic constipation.

B vitamins play a key role in intestinal health by supporting the growth and maintenance of intestinal cells, modulating the enteric neural circuitry that controls intestinal movement, regulating gut microbiota, attenuating intestinal inflammation, maintaining immune homeostasis, and influencing overall gut function. Vitamin B1 (thiamine) is an essential cofactor in the tricarboxylic acid (TCA) cycle, and its deficiency can impair TCA cycle activity, leading to a reduction in immature B cells within the gut and subsequent lymphoid atrophy—effects that can be reversed with thiamine supplementation (22). Additionally, thiamine possesses antioxidant properties, and its deficiency has been linked to oxidative stress, inflammation, neurodegeneration, and potentially, constipation (23).

Vitamin B2 (riboflavin) functions as a cofactor for redox reaction enzymes. Research by Nakano et al. demonstrated that riboflavin levels significantly impact cell cycle regulation, with deficiency impairing intestinal cell proliferation and potentially compromising mucosal integrity and gut function (24). Another study found that riboflavin supplementation reduced systemic oxidative stress in Crohn’s disease patients, exerted anti-inflammatory effects (by decreasing C-reactive protein, platelet count, erythrocyte sedimentation rate, and IL-2 levels), and mitigated clinical disease activity as measured by the Harvey-Bradshaw Index (25).

Vitamin B3, also known as niacin or nicotinamide, serves as a precursor for the synthesis of NAD and NADP, essential cofactors in numerous metabolic processes (11). Niacin functions as a pharmacological agonist of Gpr109a, promoting the anti-inflammatory properties of colonic macrophages and dendritic cells through Gpr109a signaling. This pathway facilitates the differentiation of regulatory T cells (Tregs) and IL-10-producing T cells, thereby reducing colitis and colorectal cancer risk (26). An *in vitro* study by Qi et al. demonstrated that vitamin B3 supplementation enhanced the growth rate, crypt number, and survival of intestinal organoids derived from mice (27). In a murine model, nicotinamide was found to significantly alleviate severe colitis and diarrhea, likely through activation of the mTOR signaling pathway, which controls cellular proliferation, survival, protein synthesis, and transcription, thereby modulating gut microbiota composition and reducing intestinal inflammation (28). Additionally, research by Li et al. revealed that niacin exerts protective effects in colitis-induced mice by releasing prostaglandin D2 (PGD2) and activating D-prostanoid receptor 1 (DP1), leading to decreased vascular permeability, reduced apoptosis of colonic epithelial cells, and suppression of pro-inflammatory cytokine secretion. Furthermore, a niacin-based enema was shown to promote clinical remission and mucosal healing in patients with moderate active ulcerative colitis (29). Multiple clinical trials have confirmed that enhancing NAD + levels can mitigate immune responses and reduce inflammation. Notably, increasing NAD + levels has been demonstrated to improve cardiac function (30), reduce ovarian inflammation (31), and attenuate lung inflammation caused by SARS-CoV-2 infection (32). The most direct approach to elevating NAD + concentrations involves providing its precursors to enhance endogenous synthesis. Since niacin serves as a direct NAD + precursor, its oral administration may effectively alleviate systemic inflammation, including intestinal inflammation, thereby improving gut function and reducing constipation occurrence.

Vitamin B6, also known as pyridoxal, pyridoxine, or pyridoxamine, plays a key role in the synthesis and metabolism of nucleic acids, amino acids, lipids, carbohydrates, and neurotransmitters. It contributes to intestinal immune regulation by participating in the metabolic degradation of sphingosine-1-phosphate (S1P), a lipid mediator that governs cellular trafficking and activation, including the migration of intestinal epithelial lymphocytes (33). A deficiency in vitamin B6 may disrupt intestinal mucosal immune homeostasis, leading to impaired gut function. A cross-sectional study identified a significant inverse correlation between vitamin B6 intake and the severity of irritable bowel syndrome symptoms (18). Additionally, moderate vitamin B6 supplementation has been shown to attenuate the histological and molecular markers of colitis in IBD mouse models (34). Clinical studies have reported that vitamin B6 levels are significantly lower in IBD patients relative to controls, with low plasma vitamin B6 concentrations identified as an independent risk factor for thrombosis in IBD, particularly during active disease states (35). Pyridoxal-5′-phosphate (PLP), the biologically active form of vitamin B6, has been found to exhibit an inverse relationship with multiple inflammatory markers. Low plasma PLP levels are linked to greater odds of developing cardiovascular disease (36), rheumatoid arthritis (37), IBD, and the onset of certain malignancies (38, 39). One proposed mechanism suggests that vitamin B6 is recruited to sites of inflammation, where it acts as a cofactor in metabolic pathways that generate immunomodulatory metabolites (40).

Vitamin B9, commonly known as folate, plays a critical role in cellular metabolism by serving as a one-carbon donor essential for DNA and protein synthesis, as well as methylation (41). DNA methylation is a key molecular process that influences gene expression, DNA stability, and susceptibility to mutations. A deficiency in folate can contribute to carcinogenesis by disrupting gene regulation and increasing DNA damage (42, 43). Numerous clinical studies have demonstrated an inverse association between folate intake and colorectal cancer risk (44). Furthermore, inadequate vitamin B9 levels can significantly alter immune responses; for instance, folate deficiency has been shown to suppress the activity of CD8 + T cells and NK cells, thereby reducing the body’s ability to combat infections (45). Dietary folate also plays a vital role in maintaining colonic regulatory T cells, which help prevent excessive intestinal inflammation. A folate-deficient diet has been found to selectively reduce Foxp3 + regulatory T cells in the colon, increasing susceptibility to intestinal inflammation (46). Klaassen et al. reported that worsening symptoms of Crohn’s disease (CD) are related to reduced expression of microbial genes involved in the biosynthesis of anti-inflammatory mediators, including riboflavin, thiamine, folate, and short-chain fatty acids (SCFAs), suggesting that enhancing the abundance of these mediators in the gut could offer novel therapeutic strategies for CD patients (47). Additionally, folate deficiency has been related to anemia, cancer, cognitive decline, cardiovascular disease, and developmental disorders such as neural tube defects, underscoring the necessity of dietary folate intake to maintain physiological homeostasis (48, 49).

Vitamin B12, or cobalamin, serves as a crucial cofactor in the synthesis and metabolism of DNA, RNA, proteins, lipids, and hormones. It is indispensable for red blood cell production, neurological function, and myelin formation (50). Cobalamin also acts as an immunomodulatory agent in cellular immunity. Deficiencies in vitamin B12 have been associated with reduced peripheral blood lymphocyte and CD8 + cell counts, an abnormal increase in the CD4/CD8 ratio, and impaired NK cell activity. Notably, supplementation with vitamin B12 has been shown to restore lymphocyte and CD8 + cell levels (51). Feng et al. demonstrated that vitamin B12 can mitigate excessive intestinal stem cell proliferation and inflammatory responses following injury in a fruit fly model. This protective effect appears to be mediated through the HIF-1 signaling pathway, which regulates cellular responses to low-oxygen environments. Vitamin B12 promotes mitochondrial oxygen consumption, stabilizes intestinal oxidative balance, and suppresses dextran sulfate sodium (DSS)-induced increases in Gram-negative bacteria, thereby preventing intestinal damage and barrier dysfunction. These findings were further validated in a DSS-induced mouse colitis model, confirming the protective role of vitamin B12 in maintaining intestinal epithelial integrity (52).

Choline is an essential micronutrient involved in cellular and organismal homeostasis. It serves as a precursor to acetylcholine (ACh), phospholipids, and betaine (a methyl donor), playing a pivotal role in cell membrane integrity, transmembrane signaling, methyl metabolism, neurotransmission, and the metabolism of lipids and cholesterol (11, 53). One of choline’s critical metabolites, phosphatidylcholine (PC), is a key component of intestinal mucus, and reduced PC levels have been implicated in the early stages of UC pathogenesis (54). In a fish model, Wu et al. found that choline deficiency compromises intestinal antimicrobial defense by decreasing antimicrobial component levels and promoting intestinal inflammation through increased pro-inflammatory cytokine levels together with a drop in anti-inflammatory cytokine content. Choline deficiency also weakens the intestinal physical barrier by downregulating tight junction protein mRNA expression (55). Choline metabolism intermediates, such as cytidine diphosphate (CDP)-choline, have been shown to alleviate DSS-induced colitis in mice by replenishing choline and its metabolites, activating the cholinergic anti-inflammatory pathway, modulating gut microbiota composition, and enhancing SCFA production. These effects collectively improve intestinal symptoms and histopathological abnormalities (56). Studies in pig models further support choline’s beneficial role in gut health, as dietary supplementation has been found to enhance gut microbiota diversity, upregulate expression of the anti-apoptotic gene Bcl2, suppress IL-8 expression, and promote intestinal cell proliferation (57). Additionally, choline serves as a precursor for ACh, a major neurotransmitter involved in colonic motility. Intestinal neurons communicate directly with glial cells to regulate colonic movement, with ACh playing a central role in the excitatory neural control of the circular muscle, the primary driver of intestinal propulsion. ACh binds to muscarinic receptors, inducing transient depolarization, known as excitatory junction potential. Thus, an appropriate choline supply is vital for the preservation of intestinal motility and preventing constipation (58).

Folate, vitamin B12, and choline collectively function as methyl donors critical for intestinal homeostasis. Bressenot et al. reported that a methyl-deficient diet lacking these three nutrients led to severe atrophy of the distal intestinal wall in rat pups, characterized by significantly reduced villus height and width, crypt length, crypt density, submucosal and muscular layer thickness, and enterocyte size. Furthermore, crypt apoptosis was markedly increased, and enterocyte differentiation was impaired in rats fed a methyl-deficient diet (59). In other similar reports, researchers have shown that methyl deficiency exacerbates experimental colitis in rats by enhancing oxidative stress, inhibiting apoptosis, and activating pro-inflammatory pathways including the TNF pathway, p38, cPLA2, and COX-2 (60).

Intestinal motility is regulated by a complex interplay between the gut environment, including the microbiota and their metabolites, the immune system, the enteric nervous system, and the central nervous system. Dysfunction in any of these overlapping regulatory systems can result in constipation (61). The immune system strongly regulates intestinal motility, which is modulated by myogenic, neural, and hormonal mechanisms. Immune system products have been shown to influence smooth muscle excitability, peripheral and enteric nervous system (ENS) function, and central nervous system (CNS) regulation, all of which can contribute to constipation (62). Low-grade inflammatory activity and immune activation have been observed in irritable bowel syndrome (IBS). In cases of functional constipation, elevated proportions of CD3+, CD4+, CD8+, and CD25 + T cells, increased T lymphocyte proliferation, and heightened systemic cellular immune activation have been reported. Additionally, increased levels of antibacterial antibodies, along with elevated concentrations of immunoglobulin G (IgG), immunoglobulin M (IgM), and circulating immune complexes, suggest the presence of systemic cellular and humoral immune activation in individuals with functional constipation (63).

The gut microflora is involved in both the physiological and pathological processes underlying constipation. It influences gastrointestinal functionality through interactions with the immune system, the ENS, and CNS while also modulating intestinal secretion and the hormonal environment. The microbiota and its metabolites, including SCFAs, contribute to the regulation of gastrointestinal motility, the maintenance of mucosal immune homeostasis, and intestinal fluid secretion by engaging pathways such as Toll-like receptor (TLR) signaling and the 5-hydroxytryptamine (5-HT) system. In individuals with constipation, key beneficial microbial species are suppressed, whereas the prevalence of potentially pathogenic microorganisms increases. Probiotic supplementation has been shown to modulate host physiological processes and may serve as a therapeutic approach for constipation (64). The intake of B vitamins has been demonstrated to influence gut microbiota diversity, abundance, and functionality (65). The gut microbiota not only synthesizes vitamins, providing essential micronutrients for both the host and microbial communities, but also relies on vitamins as cofactors for energy production, which can enhance bacterial metabolism, stimulate specific microbial populations, and improve their biological activity. The B-vitamin family plays a vital role in microbial interactions, metabolic processes, and signal transduction (66). For instance, thiamine is essential for the stability of gut microbiota, and its biosynthesis and transport systems are crucial for the growth of *Bacteroides thetaiotaomicron* (67). Niacin exhibits antioxidant and anti-inflammatory characteristics, regulates intestinal barrier function, and influences the production of bacterial endotoxins, thereby directly impacting the gut microflora. In a human-focused interventional study, the delivery of sustained-release microcapsules containing niacin to the ileocecal region for six weeks led to a significant increase in Bacteroidetes abundance (68). The ratio of Firmicutes to Bacteroidetes is widely regarded as a critical determinant in maintaining intestinal homeostasis (69). Vitamin B12 serves as a signaling molecule that orchestrates the spatial and functional organization of gut microbiota. Wang et al. used an *in vitro* colon model to demonstrate that cobalamin and whey supplementation enhances the proportions of Firmicutes and Bacteroidetes while reducing Proteobacteria, potentially fostering a healthier colonic environment (70). A systematic review further supports an association between vitamin B12 intake, status, or supplementation and various gut microbiome parameters, including beta-diversity, alpha-diversity, bacterial abundance, functional capacity, and SCFA biogenesis (71). Additionally, Gurwara et al. observed significant differences in gut microbiome community makeup within the colonic mucosa in response to dietary intake of riboflavin, pyridoxine, folate, and cobalamin (72). Collectively, these findings indicate that B vitamins positively influence gut microbiome composition, highlighting their potential as prebiotic agents.

Beyond their direct effects on gut microbiota, B vitamins may indirectly modulate constipation risk by improving overall dietary quality. Individuals with constipation often exhibit poor dietary patterns characterized by insufficient B-vitamin intake and lower HEI-2015 scores. Notably, higher HEI-2015 scores have been linked to a reduced risk of constipation (73). Individuals with higher HEI-2015 scores were found to consume greater amounts of fiber- and water-rich foods, such as fruit, vegetables, whole grains, and legumes. Both dietary fiber and adequate water have been demonstrated to alleviate constipation effectively (74). B vitamins are abundant in foods such as vegetables, grains, legumes, nuts, meat, and eggs, among others. Notably, vitamin B12 is synthesized by microorganisms in animal intestines, and is thus found predominantly found in foods of animal origin, such as meat and dairy products. A diversified diet incorporating these food categories can adequately fulfill the requirements for most B vitamins in healthy individuals. This suggests that increasing B-vitamin intake may not only enhance intestinal function directly but also mitigate constipation risk by promoting healthier dietary habits.

Subgroup analysis further identified potential mediators of the interplay between B-vitamin intake and constipation, especially with respect to the interaction between the consumption of alcohol and vitamin B1, folate, niacin, and choline intake. Alcohol consumption can compromise intestinal health through several mechanisms. Firstly, alcohol induces contraction of the villus core and compression of jejunal lymphatic vessels, leading to mucosal damage and impaired nutrient absorption (75, 76). Secondly, chronic alcohol intake disrupts gastric and small intestinal motility and is associated with a reduction in the proportion of nNOS-immunoreactive myenteric neurons in the mouse jejunum (77). Additionally, alcohol alters gut microbiota composition, leading to dysbiosis (78). These disruptions may exacerbate constipation symptoms, suggesting that B-vitamin supplementation could be particularly beneficial for individuals who consume alcohol regularly. These findings emphasize that lifestyle factors, such as alcohol consumption, play a crucial role in shaping the relationship between B-vitamin intake and constipation. This underscores the importance of considering individual lifestyle habits when designing intervention strategies for constipation management. Although the present analysis also suggests some variation in the link between B-vitamin intake and constipation across genders, ethnicities, education levels, diabetes and hypertension comorbidities, depression status, recreational activities, smoking, and alcohol consumption, these factors do not significantly alter the overall negative correlation trend. This supports the broad relevance of B vitamins in the prevention and management of constipation.

Despite providing valuable epidemiological insights into how B vitamins relate to constipation, this study has several limitations. Firstly, as it is based on cross-sectional data, causal relationships cannot be established. It remains unclear whether lower B-vitamin intake directly contributes to constipation or whether individuals with constipation consume less vitamin B due to irregular dietary patterns. Longitudinal studies are necessary to clarify this causal link. Secondly, both constipation status and B-vitamin intake were self-reported by participants, potentially introducing recall or reporting biases. Additionally, 24-h dietary recall may not provide an accurate reflection of long-term dietary patterns, highlighting the need for more comprehensive dietary assessment methods in future research. Finally, the data on dietary intake were obtained from the 24-h dietary recall by NHANES participants conducted prior to the interview. By assessing the types and quantities of food and beverages consumed by participants, it is possible to estimate their intake of energy, nutrients, and other food components derived from these dietary sources, including the levels of various vitamins, carbohydrates, fats, proteins, dietary fiber, minerals, and other nutrients. Additionally, other nutrients, apart from vitamin B, that were consumed by the participants may also influence constipation. Previous studies have indicated an association between higher HEI-2015 scores and reduced risk of constipation (73). Consequently, in Model 3, we adjusted for HEI-2015 scores along with other confounding factors to minimize the potential interference of additional nutrients on the research findings. Furthermore, although this study adjusted for multiple confounders, residual confounding remains a possibility. Factors such as gut microbiota composition, medication use, and underlying conditions like hypothyroidism, which can influence gut function, were not fully accounted for. Future prospective clinical trials should be performed to evaluate the therapeutic efficacy of B-vitamin supplementation in constipation management. Additionally, further study is vital to clarify the specific mechanisms through which B vitamins influence intestinal motility and to assess their effects under varying dietary and lifestyle conditions. The role of personalized dietary interventions should also be explored to optimize their practical application.

## 5 Conclusion

These findings reveal a significant negative link between B-vitamin intake and constipation, indicating that adequate consumption of B vitamins, particularly niacin, folate, and choline, may help alleviate constipation symptoms. These results offer a scientific foundation for the potential application of B vitamins in constipation management. Healthcare providers can potentially consider dietary interventions as a non-pharmacological approach to support patients affected by chronic constipation.

## Data Availability

The original contributions presented in the study are included in the article/Supplementary material, further inquiries can be directed to the corresponding author.
