# Supplementary material for: Evaluating the link between the dietary intake of vitamin B and constipation: a population-based study
Source: Front Nutr. 2025 May 29;12:1594644. doi: 10.3389/fnut.2025.1594644 (PMC12158707; doi:10.3389/fnut.2025.1594644)
Supplement: Supplementary file 1 [file Data_Sheet_1.docx]

**Supplementary Table S1:** Subgroup analyses focused on the relationship between Vitamin B1 intake and the incidence of constipation.

| Vitamin B1 | Quartile 1 | Quartile 2 | Quartile 3 | Quartile 4 | p for trend | p for interaction |
| --- | --- | --- | --- | --- | --- | --- |
| Sex |  |  |  |  |  | 0.13 |
| Female | ref | 0.88(0.70,1.11) | 0.65(0.47,0.91) | 0.83(0.58,1.21) | 0.08 |  |
| Male | ref | 0.69(0.41,1.16) | 0.42(0.24,0.72) | 0.44(0.26,0.76) | 0.002 |  |
| Race |  |  |  |  |  | 0.05 |
| Mexican American | ref | 1.09(0.67,1.77) | 0.50(0.29,0.85) | 0.59(0.34,1.01) | 0.004 |  |
| Non-Hispanic Black | ref | 0.66(0.43,1.03) | 0.49(0.30,0.81) | 0.69(0.39,1.19) | 0.1 |  |
| Non-Hispanic White | ref | 0.90(0.65,1.25) | 0.58(0.37,0.90) | 0.51(0.35,0.76) | <0.001 |  |
| Other Hispanic | ref | 0.91(0.52,1.57) | 0.92(0.35,2.45) | 0.57(0.25,1.29) | 0.22 |  |
| Other Race | ref | 0.18(0.06,0.56) | 0.14(0.05,0.39) | 0.29(0.09,0.88) | 0.05 |  |
| Educational status |  |  |  |  |  | 0.25 |
| Less than high school | ref | 0.81(0.50,1.32) | 0.28(0.09,0.85) | 0.29(0.15,0.57) | <0.001 |  |
| High school | ref | 0.68(0.47,0.99) | 0.49(0.31,0.80) | 0.57(0.37,0.90) | 0.02 |  |
| More than high school | ref | 0.99(0.67,1.45) | 0.64(0.44,0.92) | 0.53(0.36,0.80) | <0.001 |  |
| Diabetes mellitus |  |  |  |  |  | 0.62 |
| No | ref | 0.82(0.65,1.02) | 0.51(0.37,0.72) | 0.49(0.35,0.68) | <0.0001 |  |
| Yes | ref | 0.61(0.39,0.94) | 0.47(0.25,0.89) | 0.53(0.32,0.90) | 0.01 |  |
| Hypertension |  |  |  |  |  | 0.71 |
| No | ref | 0.78(0.57,1.06) | 0.49(0.32,0.73) | 0.52(0.36,0.77) | <0.001 |  |
| Yes | ref | 0.80(0.54,1.19) | 0.54(0.37,0.81) | 0.42(0.28,0.62) | <0.0001 |  |
| Depression |  |  |  |  |  | 0.48 |
| No | ref | 0.86(0.69,1.07) | 0.55(0.39,0.78) | 0.55(0.40,0.75) | <0.0001 |  |
| Yes | ref | 0.57(0.34,0.98) | 0.43(0.22,0.86) | 0.34(0.15,0.77) | 0.004 |  |
| Recreational activity |  |  |  |  |  | 0.79 |
| No | ref | 0.74(0.55,1.00) | 0.53(0.38,0.75) | 0.50(0.33,0.74) | <0.001 |  |
| Yes | ref | 0.90(0.65,1.26) | 0.51(0.32,0.81) | 0.54(0.38,0.77) | <0.001 |  |
| Smoking status |  |  |  |  |  | 0.25 |
| No | ref | 0.73(0.56,0.95) | 0.53(0.38,0.73) | 0.45(0.33,0.61) | <0.0001 |  |
| Yes | ref | 1.00(0.54,1.86) | 0.40(0.21,0.76) | 0.65(0.35,1.18) | 0.05 |  |
| Drinking status |  |  |  |  |  | 0.004 |
| No | ref | 1.16(0.84,1.61) | 0.72(0.49,1.05) | 0.91(0.63,1.33) | 0.2 |  |
| Yes | ref | 0.65(0.48,0.88) | 0.44(0.30,0.65) | 0.37(0.25,0.56) | <0.0001 |  |

**Supplementary Table S2:** Subgroup analyses focused on the relationship between niacin intake and the incidence of constipation.

| Niacin | Quartile 1 | Quartile 2 | Quartile 3 | Quartile 4 | p for trend | p for interaction |
| --- | --- | --- | --- | --- | --- | --- |
| Sex |  |  |  |  |  | 0.74 |
| Female | ref | 0.92(0.69,1.22) | 0.63(0.48,0.83) | 0.65(0.47,0.90) | <0.001 |  |
| Male | ref | 1.01(0.62,1.65) | 0.53(0.35,0.79) | 0.67(0.45,0.98) | 0.02 |  |
| Race |  |  |  |  |  | 0.9 |
| Mexican American | ref | 0.88(0.57,1.36) | 0.52(0.27,0.98) | 0.56(0.31,1.01) | 0.03 |  |
| Non-Hispanic Black | ref | 1.02(0.75,1.40) | 0.47(0.29,0.78) | 0.57(0.37,0.88) | 0.002 |  |
| Non-Hispanic White | ref | 0.86(0.59,1.25) | 0.51(0.37,0.72) | 0.47(0.34,0.63) | <0.0001 |  |
| Other Hispanic | ref | 1.10(0.54,2.26) | 0.85(0.41,1.76) | 0.32(0.17,0.59) | <0.001 |  |
| Other Race | ref | 0.76(0.15,3.94) | 0.45(0.10,2.12) | 0.35(0.07,1.71) | 0.14 |  |
| Educational status |  |  |  |  |  | 0.18 |
| Less than high school | ref | 0.95(0.55,1.64) | 0.19(0.10,0.36) | 0.37(0.20,0.68) | <0.0001 |  |
| High school | ref | 0.85(0.61,1.19) | 0.49(0.35,0.69) | 0.48(0.36,0.66) | <0.0001 |  |
| More than high school | ref | 0.96(0.62,1.49) | 0.66(0.45,0.96) | 0.53(0.37,0.77) | <0.0001 |  |
| Diabetes mellitus |  |  |  |  |  | 0.13 |
| No | ref | 0.96(0.73,1.26) | 0.54(0.42,0.68) | 0.49(0.38,0.64) | <0.0001 |  |
| Yes | ref | 0.51(0.28,0.90) | 0.37(0.20,0.69) | 0.29(0.16,0.53) | <0.0001 |  |
| Hypertension |  |  |  |  |  | 0.67 |
| No | ref | 0.89(0.66,1.20) | 0.49(0.37,0.64) | 0.49(0.35,0.67) | <0.0001 |  |
| Yes | ref | 0.85(0.57,1.26) | 0.53(0.36,0.79) | 0.39(0.30,0.52) | <0.0001 |  |
| Depression |  |  |  |  |  | 0.31 |
| No | ref | 0.96(0.70,1.32) | 0.57(0.43,0.77) | 0.50(0.37,0.66) | <0.0001 |  |
| Yes | ref | 0.69(0.37,1.28) | 0.30(0.16,0.56) | 0.48(0.26,0.90) | 0.005 |  |
| Recreational activity |  |  |  |  |  | 0.49 |
| No | ref | 0.91(0.67,1.23) | 0.46(0.34,0.63) | 0.44(0.33,0.58) | <0.0001 |  |
| Yes | ref | 0.86(0.52,1.44) | 0.62(0.41,0.93) | 0.56(0.39,0.79) | <0.001 |  |
| Smoking status |  |  |  |  |  | 0.11 |
| No | ref | 0.75(0.57,0.98) | 0.50(0.38,0.65) | 0.43(0.32,0.58) | <0.0001 |  |
| Yes | ref | 1.49(0.78,2.85) | 0.51(0.25,1.03) | 0.59(0.34,1.04) | 0.002 |  |
| Drinking status |  |  |  |  |  | 0.04 |
| No | ref | 1.04(0.75,1.44) | 0.77(0.56,1.04) | 0.72(0.52,0.98) | 0.005 |  |
| Yes | ref | 0.80(0.57,1.14) | 0.42(0.30,0.58) | 0.40(0.29,0.54) | <0.0001 |  |

**Supplementary Table S3:** Subgroup analyses focused on the relationship between choline intake and the incidence of constipation.

| Choline | Quartile 1 | Quartile 2 | Quartile 3 | Quartile 4 | p for trend | p for interaction |
| --- | --- | --- | --- | --- | --- | --- |
| Sex |  |  |  |  |  | 0.26 |
| Female | ref | 0.74(0.58,0.95) | 0.69(0.56,0.85) | 0.73(0.55,0.98) | 0.01 |  |
| Male | ref | 0.95(0.57,1.60) | 0.78(0.49,1.23) | 0.61(0.41,0.91) | 0.01 |  |
| Race |  |  |  |  |  | 0.42 |
| Mexican American | ref | 1.42(0.85,2.35) | 0.89(0.47,1.70) | 0.88(0.46,1.66) | 0.31 |  |
| Non-Hispanic Black | ref | 0.54(0.35,0.85) | 0.46(0.28,0.74) | 0.50(0.32,0.77) | 0.01 |  |
| Non-Hispanic White | ref | 0.70(0.51,0.95) | 0.62(0.47,0.83) | 0.43(0.30,0.62) | <0.0001 |  |
| Other Hispanic | ref | 0.79(0.48,1.29) | 0.76(0.39,1.49) | 0.71(0.40,1.25) | 0.29 |  |
| Other Race | ref | 0.84(0.30,2.36) | 0.40(0.09,1.76) | 0.27(0.08,0.97) | 0.03 |  |
| Educational status |  |  |  |  |  | 0.92 |
| Less than high school | ref | 0.75(0.42,1.34) | 0.82(0.44,1.55) | 0.46(0.23,0.93) | 0.05 |  |
| High school | ref | 0.69(0.48,1.01) | 0.54(0.39,0.76) | 0.44(0.30,0.63) | <0.0001 |  |
| More than high school | ref | 0.79(0.56,1.10) | 0.66(0.43,1.03) | 0.52(0.36,0.76) | <0.001 |  |
| Diabetes mellitus |  |  |  |  |  | 0.92 |
| No | ref | 0.73(0.57,0.95) | 0.61(0.46,0.80) | 0.47(0.36,0.60) | <0.0001 |  |
| Yes | ref | 0.62(0.35,1.07) | 0.48(0.26,0.91) | 0.39(0.21,0.71) | 0.002 |  |
| Hypertension |  |  |  |  |  | 0.34 |
| No | ref | 0.77(0.55,1.09) | 0.55(0.37,0.83) | 0.50(0.37,0.69) | <0.0001 |  |
| Yes | ref | 0.62(0.43,0.90) | 0.65(0.44,0.97) | 0.37(0.26,0.53) | <0.0001 |  |
| Depression |  |  |  |  |  | 0.76 |
| No | ref | 0.74(0.57,0.95) | 0.62(0.48,0.81) | 0.50(0.38,0.65) | <0.0001 |  |
| Yes | ref | 0.78(0.37,1.64) | 0.55(0.25,1.22) | 0.35(0.19,0.64) | 0.003 |  |
| Recreational activity |  |  |  |  |  | 0.9 |
| No | ref | 0.76(0.56,1.03) | 0.63(0.46,0.85) | 0.46(0.34,0.63) | <0.0001 |  |
| Yes | ref | 0.67(0.45,1.01) | 0.56(0.38,0.84) | 0.47(0.33,0.69) | <0.001 |  |
| Smoking status |  |  |  |  |  | 0.96 |
| No | ref | 0.74(0.57,0.97) | 0.60(0.45,0.79) | 0.47(0.35,0.61) | <0.0001 |  |
| Yes | ref | 0.62(0.29,1.32) | 0.56(0.34,0.93) | 0.43(0.25,0.76) | 0.005 |  |
| Drinking status |  |  |  |  |  | 0.02 |
| No | ref | 0.84(0.57,1.23) | 0.85(0.63,1.14) | 0.77(0.57,1.02) | 0.1 |  |
| Yes | ref | 0.66(0.48,0.91) | 0.50(0.36,0.69) | 0.38(0.28,0.52) | <0.0001 |  |
